# Incidence and risk factors for medication‐related osteonecrosis after tooth extraction in cancer patients—A systematic review

**DOI:** 10.1002/cre2.698

**Published:** 2022-12-04

**Authors:** Nurda Schwech, Johanna Nilsson, Pia Gabre

**Affiliations:** ^1^ Department of Orofacial Medicine, Public Dental Health Uppsala County Council Uppsala Sweden; ^2^ Department of Plastic & Oral and Maxillofacial Surgery, Department of Surgical Sciences Uppsala University Uppsala Sweden; ^3^ Department of Oral & Maxillofacial Surgery Zealand University Hospital Roskilde Denmark; ^4^ Department of Cariology, Institute of Odontology The Sahlgrenska Academy of Gothenburg Gothenburg Sweden

**Keywords:** medication‐related osteonecrosis, risk factors, tooth extractions

## Abstract

**Objectives:**

Antiresorptive medication has been reported to be associated with medication‐related osteonecrosis of the jaw (MRONJ). This systematic review aims at investigating the incidence of and risk factors for MRONJ after tooth extractions in cancer patients treated with high‐dose bisphosphonate and denosumab (BP and DS).

**Material and Methods:**

The protocol followed the PRISMA statement list and was registered in PROSPERO. Searches were performed for literature published up to April 2021 in the electronic databases PubMed, Embase, Web of Science, and CINAHL and then supplemented by manual research.

**Results:**

The search process resulted in 771 identified articles, of which seven studies fitted the population, intervention, comparison, and outcome framework. All were observational studies and four had control groups. A total of 550 patients treated with BP and DS were identified of whom 271 had received tooth extractions after medication onset. Due to significant heterogenicity in the collected data, only a qualitative analysis was performed. The MRONJ incidence after tooth extractions varied between 11% and 50% at the patient level. MRONJ occurred up to 3 years after the tooth extraction. Teeth affected by inflammation before the extraction and additional osteotomy during the surgical procedure were identified as risk factors.

**Conclusions:**

Reliable methods of diagnosing MRONJ and adequate follow‐up periods are important factors in obtaining the actual incidence of MRONJ after tooth extractions in patients treated with high‐dose BP and DS.

## 1 INTRODUCTION

Medication‐related osteonecrosis of the jaw (MRONJ) is an umbrella term for all osteonecrosis associated with medicines. Bisphosphonates (BPs) and denosumab (DS) are antiresorptive medications used in the treatment of cancer, osteoporosis, Paget's disease, and other bone diseases, which have been reported to be related to the serious side‐effect osteonecrosis of the jaw (Ruggiero et al., 2014). Although the condition is uncommon, a higher proportion of osteonecrosis has been reported when using high‐dose medicines in the treatment of cancer as compared to osteoporosis, which is treated with low‐dose BP (Kühl et al., 2012). At the same time, BP and DS as adjuvant therapy have the potential to improve the quality of life for patients with advanced cancer (Ruggiero et al., 2022). The diagnosis is based on clinical and radiological findings and is defined as exposed, necrotic bone in the maxillofacial region that can be probed through an intraoral or extraoral fistula(e) that has persisted for more than 8 weeks in patients receiving an antiresorptive medication alone or in combination with immune modulators or antiangiogenic medications without a history of radiation therapy to the jaws (Ruggiero et al., 2022). Another category is nonexposed osteonecrosis, which accounts for 25% of cases (Fedele et al., 2015). Consensus on diagnosis criteria is not obvious but a common classification is defined in the AAOMS position paper, in which four stages are described (Ruggiero et al., 2022).

The first report on MRONJ was published by Marx in 2003 (Marx, 2003). The complete etiology of MRONJ has not yet been fully understood but several theories have been presented. Since the remodeling of bone is considerably higher in the jaw than in other skeletal parts, one theory is that BP suppresses the remodeling process, which leads to serious damage in the jaws (Russell et al., 2007). Another theory is that a dental infection causes MRONJ, which could explain why patients with periodontitis are more often diagnosed with the condition (Aghaloo et al., 2011). The overall incidence of MRONJ is reported in several studies, with results varying widely from 0.01% after oral use of low‐dose BP (Ulmner et al., 2014) to 14.4% in patients who received high‐dose intravenous medication (Coello‐Suanzes et al., 2018). Patients with a cancer diagnosis treated with high‐dose zoledronic acid have been reported to have a cumulative incidence of MRONJ between 0.7% and 6.7%, while patients treated with DS showed a risk between 0.7% and 3.64% (Ruggiero et al., 2014). In a Swedish prospective cohort study, 55 consecutive patients were examined during the years 2012–2015. The incidence of MRONJ was 0.043% among patients treated with low‐dose oral BP, 1.03% among those on high‐dose intravenous BP, and 3.64% in patients treated with high‐dose DS (Hallmer et al., 2018). The same research group has recently published a prospective study of patients with breast cancer with a follow‐up period of 77 months. Patients treated with high‐dose DS had three times higher frequency of MRONJ compared with zoledronic acid (13.6% and 4.1%, respectively; Hallmer et al., 2020). A longer treatment time for the high‐dose antiresorptive drug and high dosage increased the risk for MRONJ (Otto et al., 2015; Ruggiero et al., 2014). In a systematic review with a meta‐analysis including 12 studies, the prevalence of MRONJ for sequential BP‐DS therapy was reported to be 13% in cancer patients treated with high‐dose medication, while for BP and DS, only 5% and 4%, respectively (Srivastava et al., 2021).

Risk factors are an important explanation for the large differences in the incidence of MRONJ. The most common trigger factor is tooth extractions, especially when teeth have severe inflammation conditions (Hasegawa et al., 2019). Other suggested risk factors are diabetes, immunosuppressive conditions, and anemia (Ruggiero et al., 2014). Several other risk factors have been suggested such as age, smoking, obesity, and the use of other additional drugs, for example, corticosteroids and chemotherapy. The clinician is often faced with difficult decisions when teeth need to be extracted in patients on antiresorptive medication. Could the surgical procedure hurt the patient more than refraining from treatment? Although it is known that an extraction poses a risk for MRONJ, most extractions seem to be performed without complications (Hasegawa et al., 2019; Yamazaki et al., 2012). Knowledge of how often MRONJ occurs after extractions in patients on antiresorptive medication is limited. Thus, the aim of the present study was to, in a systematic review, assess the incidence of bone necrosis (MRONJ) after tooth extractions in patients suffering from cancer and treated with high‐dose BP and DS. Further aims were to identify risk factors for developing MRONJ after tooth extraction such as demographic factors, oral health status, comorbidity, and other drug use, as well as a choice of surgical processes and preventive measures.

## 2 MATERIALS AND METHODS

The research process followed the PRISMA statement checklist (Moher et al., 2009). The systematic review was registered on the International Prospective Register of Systematic Reviews (PROSPERO, protocol number CRD42021239321).

A literature search was conducted to find studies that reported or allowed a calculation of the incidence of MRONJ after tooth extractions in patients treated with BP and DS on cancer indication. The eligibility criteria were based on the PICO strategy: population, intervention, comparison, and outcome:

(1) The population were men and women suffering from cancer and treated with high dose BP and DS and 18 years or older.
(2) The intervention was tooth extractions, regardless of the extraction technique, during BP and DS therapy.
(3) The comparison was tooth extraction resulting in MRONJ compared to those not leading to MRONJ.
(4) The primary outcome was MRONJ after tooth extractions as described by AAOMS (Ruggiero et al., 2014) or other structured diagnostic criteria.

In addition, factors that were reported to prevent, reduce or increase the occurrence of MRONJ in conjunction with tooth extractions were registered. Randomized controlled studies, controlled studies without randomization, and other types of studies such as cohort studies, observation studies, and qualitative studies were included in the search. Both retrospective and prospective study designs were accepted. No restriction regarding the date of publication or publication status was used. The search included articles published in English or Swedish available in full text. Exclusion criteria were studies published in other languages than English and Swedish, systematic and nonsystematic reviews, publications not available in full text, animal studies, and case reports with less than eight patients. In addition, studies where patients had, in addition to treatment with BP and DS, received radiotherapy involving the jaw were excluded.

### 2.1 Search strategy

A librarian conducted a comprehensive systematic literature search in April 2021. A combination of descriptors was identified using Medical Subject Headings (MeSH) terms and related terms in the electronic bibliography databases PubMed (NLM), Embase, Web of Science, and CINAHL. Reference lists of certain studies were hand‐searched. Unpublished studies were searched on the trial registry ClinicalTrials.

The search strategy used the following keywords: Antiresorptive therapy; Antiresorptive drugs; Bisphosphonate; Osteonecrosis of the jaw; Cancer patients; Tooth extraction; Medication‐related osteonecrosis of the jaw; Preventive measures; Gentle extraction technique; Antibiotics; Bone‐modifying agents; Anti‐neoplastic agents; Bone density conservative agents; MRONJ; BRONJ; Bone resorption; Bone necrosis; Diphosphonates; Denosumab; Zoledronic acid; Zoledronate; Pamidronate; Ibandronic acid; Clodronate; Dental management; Risk factors. As an example of the search process, the search string for the database PubMed is shown in Supporting Information: Appendix, Figure A.

**Figure 1:**
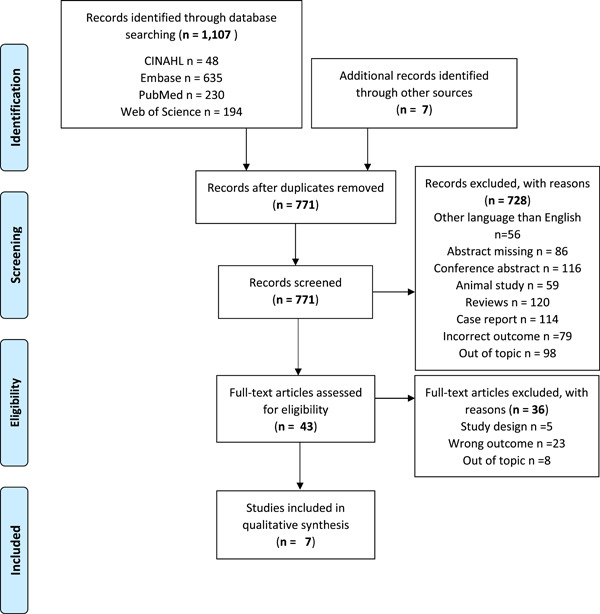
Flow chart of the selection process

The identified studies were assessed in two steps and the predetermined criteria for inclusion and exclusion were used in both. In the first step, a pre‐selection was made based on screening of titles and abstracts and those studies that did not meet the eligibility criteria were excluded. Two independent reviewers (NS and PG) performed the screening, and in case of doubt of eligibility, a consensus discussion took place. A third reviewer (JN) was to be consulted to resolve any disagreement. The two reviewers were trained and calibrated by assessing articles together before the independent assessment started. The process was repeated in phase two, but now the articles were read in full text. The assessment was still independent, with a third reviewer involved if needed. The selection process is shown in Figure 1.

Data collection forms were developed, and all studies assessed in full text, both excluded and included, were independently registered by the two reviewers along with the characteristics of the studies. Finally, data from the included studies were collected in a Microsoft Excel spreadsheet and converted into the tables shown in the Result section. In case of uncertainties, the reviewers compared and discussed the findings and, if needed, re‐examined the results until a consensus was reached. The following information was independently extracted from the studies: authors, publication year, the country in which the study was conducted, number of involved institutions, study design, time period for data registration, sample size, participants' age and gender, number of extractions, type of medication, duration of treatment, follow‐up period, clinical MRONJ presentation, the proportion of MRONJ after extractions and risk factors.

### 2.2 Quality assessment

**Figure 2:**
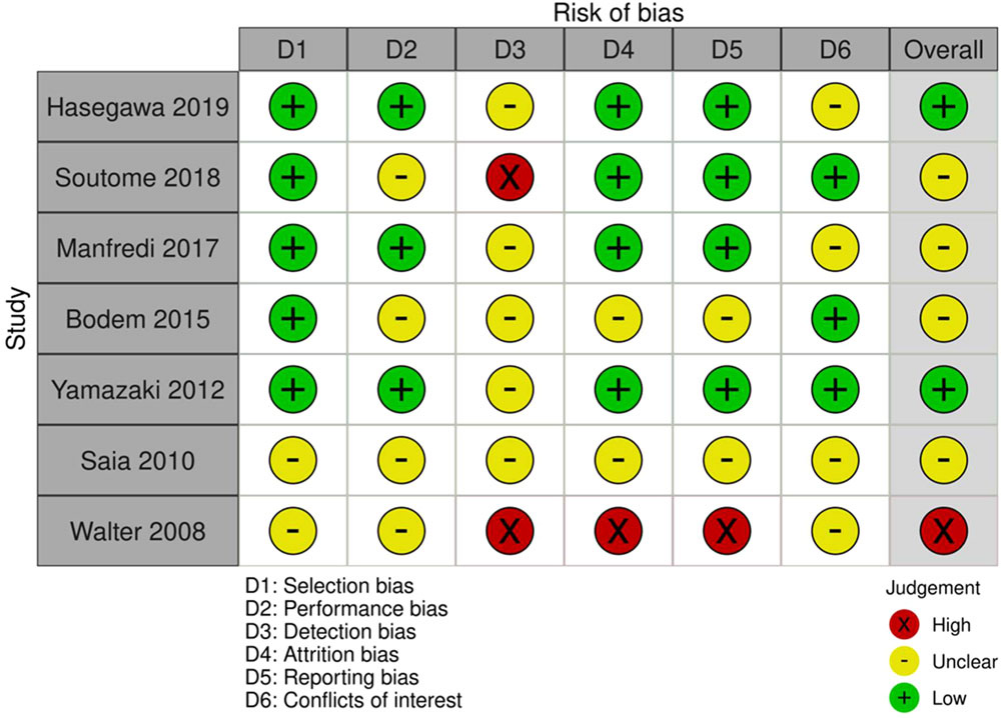
Risk of bias summary: review of authors' judgment of risk bias items for each study in the review

Using checklists adapted to different study designs from the Swedish Council on Technology Assessment in Health Care (SBU, 2018), the included articles were critically appraised according to their scientific quality. Guided by the checklists, the two reviewers independently assessed each study. If disagreement arose, the outcome was discussed until a consensus was reached. The quality criteria assessed sources of error (bias) in the domain's selection and performance, and the detection and attrition bias were estimated per outcome measure. In addition, reporting bias and conflict of interest were covered. Each question was answered on a four‐grade scale (yes, no, unclear and nonapplicable). Based on the results of each section, a summary quality assessment of high, moderate, or low was then assigned to each study (SBU, 2018). The domains and quality grading are shown in Figure 2.

### 2.3 Summary measures and synthesis of results

Due to different types of comparators, ranges of outcome measures, and different follow‐up periods, the heterogenicity was estimated to be too high for performing a meta‐analysis. Instead, a narrative synthesis is provided in text, tables, and figures.

The quality of the evidence for the outcomes was judged according to GRADE (Grading of Recommendations Assessment, Development, and Evaluation; Balshem et al., 2011). The criteria assessed limitations, consistency, directness, precision, publication bias, and magnitude of effect. The evidence was summarized in one of the categories high, moderate, low, or very low.

## 3 RESULTS

### 3.1 Study selection

A total of 771 articles were identified in the literature search (Figure 1). In the process of assessing the abstracts, 672 articles were excluded. Systematic and nonsystematic reviews were one of the exclusion criteria and this category became the most common reason for exclusion (of the 120 excluded review articles, 97 were nonsystematic). The remaining 43 articles were read in full text and 7 of these articles were found to meet the inclusion criteria (Bodem et al., 2015; Hasegawa et al., 2019; Manfredi et al., 2017; Saia et al., 2010; Soutome et al., 2018; Walter et al., 2008; Yamazaki et al., 2012). Reasons for exclusions are described in Figure 1 and the articles deselected in the last stage of the process are shown in Supporting Information: Appendix, Table 1. In the last selection step, two of three studies were excluded because MRONJ was not reported in such a way that the incidence after extractions could be calculated, that is, the outcome did not meet PICO. The study design, characteristics of participants, and the main results in the included studies are presented in Table 1.

**Table 1:** Study design and patient characteristics in included studies

Study	Number of patients	Mean age year (±SD)	Number of extractions	Type of BP/DS	Duration of treatment	Outcome MRONJ after tooth extraction
design				No of patients		
Data period	Sex	Range		(%)	Follow‐up	
Hasegawa et al. (2019)	85, all had extractions	64.5 (±11.5)	163 teeth	ZA *n* = 46 (54)	Mean 14.7 mo. (±13.8)	41 teeth (25.2%)
		Range 39–90		DS *n* = 31 (37)		25 patients (29%)
Retrospective 2008–2016	Female 51			ZA/DS *n* = 6 (7)	Range 1–60	Mandible 75.6%
	Male 34			AD/DS *n* = 1 (1)	<8 mo. *N* = 59	Maxilla 24.4%
				RD/DS *n* = 1 (1)	≥8 mo. *N* = 95	
					Follow‐up unclear	
Soutome et al. (2018)	135, 60 had extractions	62.6 SD unknown	21 patients after BP/DS‐start	ZA *n* = 80 (59)	Unclear follow‐up 3000 days. Exclusion: <6 mo.	6 patients (28.6%)
				DS *n* = 55 (41)		
Retrospective with control group 2011– July 2017	Male 64					
	Female 71					
Manfredi et al. (2017)	156, 50 had extractions	69.0 (±11.2)	20 patients after BP/DS‐start	ZA *n* = 156 (100)	Mean no. of treatments 22.3–23.4	8 patients (40%)
Retrospective with control group 2004–2010	Male 38	No MRONJ			Follow‐up 60 mo.	
	Female 118	64.6 (±15.1)				
Bodem et al. (2015)	61, all had extractions	65.7 (±12.7)	184 teeth	ZA (62.4)	Mean 40.3 mo.	10 teeth (5.4%)
		Range 34–87	102 sites	ID (28.3)	(SD ± 32.9)	8 patients (13.1%)
Prospective	Male 19			PD (9.3)	Range 4–245	Mandible 50%
Period not shown	Female 42				Follow‐up ≥12 w.	Maxilla 50%
Saia et al. (2010)	44, all had extractions	65 (SD not shown)	185 teeth	ZA *n* = 38 (63.3)	Unknown duration of high‐dose BP/DS	5 patients (11.4%)
				PA *n* = 24 (40)		
Prospective	Male 18	Range 17–84		NA *n* = 4 (6.7)	Drug holiday 1–3 mo.	
March 2006–2008	Female 42			RD *n* = 2 (3)	Follow‐up 12 mo.	
Walter et al. (2008)	43, 14 had extractions	70.2 (±5.8)	14 patients	ZA *n* = 38 (88.4)	When examined: mean no. of BP/DS applications: 21.4 (±15.1), range 1–59	7 patients (50%)
		Range not shown		ZA + BD *n* = 2 (4.7)		
Cross sectional with control group July 2006–October 2007	Male 43			ZA + PD *n* = 3 (7)	All had BP/DS ≥ 14 times	
	Female 0					
Yamazaki et al. (2012)	26 with high‐dose BP	Median 65 range unknown	26 patients	IN *n* = 1 (3.8)	Median 8.4 mo.	4 patients (14.8%)
				PD *n* = 3 (11.5)	Range 1–96 mo.	
Retrospective with control group April 2006–June 2009	Women 63% Controls, no: BP: 3090, Women 51.8%	Controls: median 39, range 20–94		ZA *n* = 13 (50)	Follow‐up 42 mo.	
				IN + PD *n* = 2 (7.7)		
				PD + ZA *n* = 7 (26.9)		

Abbreviations: AD, Alendronate; BD, Bondronate; DS, Denosumab; ID, Ibandronate; IN, Incadronate; Mo, months; ND, Neridronate; PD, Pamidronate; RD, Risedronate; ZA, Zoledronate.

Of the seven selected articles, four had a retrospective study design (Hasegawa et al., 2019; Manfredi et al., 2017; Soutome et al., 2018; Yamazaki et al., 2012), two had a prospective and longitudinal design (Bodem et al., 2015; Saia et al., 2010) and one had a cross‐sectional design (Walter et al., 2008). Three studies had a control group consisting of patients on high‐dose BP or DS but without having undergone tooth extractions (Manfredi et al., 2017; Soutome et al., 2018; Walter et al., 2008), while the control group in the study by Yamazaki et al. (2012) consisted of a population with completed extractions but not using antiresorptive medication. Three studies were performed in Japan (Hasegawa et al., 2019; Soutome et al., 2018; Yamazaki et al., 2012), two in Italy (Manfredi et al., 2017; Saia et al., 2010), and two in Germany (Bodem et al., 2015; Walter et al., 2008). A total of 3640 patients, of which 3090 represent the control group in the study by Yamazaki et al. (2012) were included. Three of the studies included patients from one institution (Bodem et al., 2015; Manfredi et al., 2017; Yamazaki et al., 2012). Three studies brought participants from two institutions (Saia et al., 2010; Soutome et al., 2018; Walter et al., 2008) and one study from 10 institutions (Hasegawa et al., 2019). All included studies used the AAOMS criteria in the definition of MRONJ (Ruggiero et al., 2022).

### 3.2 Tooth extractions

The main research question in this study was to investigate the incidence of MRONJ after tooth extractions performed after the onset of BP and DS treatment. Consequently, all included studies showed this outcome. All studies showed the incidence at the patient level, while two studies showed the incidence at both patient and tooth levels. The two articles reporting at the specific tooth level showed an MRONJ incidence after extraction of 5.4% and 25.2% (Bodem et al., 2015; Hasegawa et al., 2019). The incidence of MRONJ after tooth extraction related to the patient level varied between 11.4% and 50% (Table 1). The control groups with patients who received BP and DS but no extraction showed an incidence of MRONJ of 3.4%–9.3% (Manfredi et al., 2017; Soutome et al., 2018; Walter et al., 2008). In the population with no antiresorptive medication treatment, but extractions, the prevalence of osteonecrosis was 0.03% (Yamazaki et al., 2012; Table 1). In four of the studies, the clinical stage of MRONJ (Ruggiero et al., 2022) was reported for a total of 46 patients (Bodem et al., 2015; Hasegawa et al., 2019; Manfredi et al., 2017; Saia et al., 2010). Of these, 6.5% were assessed as stage 0, 50% as stage 1, 32.6% as stage 2, and 10.9% as stage 3.

### 3.3 Risk factors

According to Bodem et al. (2015), all extractions were performed using a standardized surgical extraction protocol as follows: (I) elevation of a mucosal flap with bilateral release incisions, if necessary; (II) osteotomy, if necessary; (III) extraction of the teeth; and (IV) tension‐free closure of the alveolar socket. MRONJ was more common if an additional osteotomy was performed on the mandible, 33%, compared with 7.3% when no osteotomy was necessary (Table 2). Hasegawa et al. (2019) studied the use of any additional surgical procedure, and root amputation was the only factor that increased the risk of MRONJ (Table 2).

**Table 2:** Investigated potential factors (marked with X) that could have affected the occurrence of MRONJ in included studies. Statistically significant differences in results marked with elevated letters a–m

Risk factors	Walter et al. (2008)	Saia et al. (2010)	Yamazaki et al. (2012)	Bodem et al. (2015)	Manfredi et al. (2017)	Soutome et al. (2018)	Hasegawa et al. (2019)
Age	X	X	X	X	X	X	X
Gender	X	X	X	X	X	X^a^	X
Tobacco use	–	–	X	–	X	X	X
Alcohol use	–	–	X	–	–	–	–
Antibiotics preoperative	–	X	X	X	X	–	X
Choice of medication	X	X	X	X	X	–	X
Duration	–	–	–	–	–	–	X^b^
Immunosuppr. therapy	–	–	–	–	–	–	X^c^
Corticosteroids	–	–	X	X	–	–	–
Chemotherapy	X	–	X	–	–	–	X
Diabetes	X	–	X	–	–	X	X
Type of cancer	–	X	–	X	X	–	–
Prevalence of inflammation	–	X^d^	X^e^	–	X^f^	X^g^	X^h^
Preventive measures	X	–	–	–	X^i^	X^j^	X
Extraction protocol	–	–	–	X^k^	–	–	X^l^
Extraction site	–	–	–	X	X	–	X^m^

^a^ Being female, *p* = .033.

^b^ Duration of medication ≥ 8 months, <.001.

^c^ Immunosuppressive therapy, *p* = .049.

^d^ Tooth extraction when baseline osteomyelitis was present, *p* < .0001.

^e^ Alveolar bone loss score, *p* = .022.

^f^ Severe periodontal disease, *p* = .025.

^g^ Teeth with clinical infection symptoms, *p* = .001.

^h^ Teeth with pre‐existing inflammation, *p* < .001.

^i^ Starting the preventive dental program after the onset of medication therapy, *p* = .02.

^j^ BP/DS administration for more than 6 months before the first visit to the dental unit, *p* = .027.

^k^ Additional osteotomy in the mandible during tooth extractions, *p* = .027.

^l^ Root amputation, *p* < .001.

^m^ Extraction of the mandibular tooth, *p* < .001.

No associations could be found between preoperatively administered antibiotics and the incidence of MRONJ (Bodem et al., 2015; Hasegawa et al., 2019; Manfredi et al., 2017; Saia et al., 2010; Yamazaki et al., 2012). Hasegawa et al. (2019) reported that 76% of the teeth were situated in the mandible compared with 24% in the maxilla (Table 2).

None of the studies found any statistically significant age differences between subjects suffering from MRONJ and others, while one study reported being a woman as a predisposing factor (Soutome et al., 2018). Neither tobacco nor alcohol use showed associations with MRONJ (Hasegawa et al., 2019; Manfredi et al., 2017; Soutome et al., 2018; Yamazaki et al., 2012; Table 2).

A higher risk for MRONJ was reported to be associated with signs of infection already before the extraction (Hasegawa et al., 2019) such as severe periodontal disease (Manfredi et al., 2017), clinical symptoms (Soutome et al., 2018), alveolar bone loss score (Yamazaki et al., 2012) and osteomyelitis (Saia et al., 2010; Table 2). In the study by Manfredi et al. (2017), patients were advised to undergo a dental check every 4 months and a professional oral hygiene recalls every 6–12 months including instructions for daily home oral hygiene procedures. Starting the preventive dental program after the beginning of BP therapy involved a greater risk for MRONJ (Table 2). Soutome et al. (2018) reported that the risk of bone necrosis increased when BP or DS had been administered more than 6 months before the first visit to the dental unit (Table 2).

The choice of medication was analyzed in six of the articles (Bodem et al., 2015; Hasegawa et al., 2019; Manfredi et al., 2017; Saia et al., 2010; Walter et al., 2008; Yamazaki et al., 2012). No statistically significant difference between the different drug compositions could be found. Hasegawa et al. (2019) reported a higher risk when the medication had been ongoing for 8 months or more (Table 2). None of the studies could report significant associations between doses of BP and DS and MRONJ. The mode of administration of the medication influenced the risk of MRONJ. The risk ratio for high‐dose intravenous medication compared with low‐dose oral administration was 14.6 (95% confidence interval 1.7–125.8; Yamazaki et al., 2012). Hasegawa et al. (2019) demonstrated an increased risk of MRONJ during immunosuppressive therapy. Contrarily, no statistically significant differences could be seen with the use of corticosteroids (Bodem et al., 2015; Yamazaki et al., 2012) and chemotherapy (Hasegawa et al., 2019; Walter et al., 2008; Yamazaki et al., 2012; Table 2) nor could connections between MRONJ and diabetes (Hasegawa et al., 2019; Soutome et al., 2018; Walter et al., 2008; Yamazaki et al., 2012) or between the type of cancer and bone necrose be found (Bodem et al., 2015; Manfredi et al., 2017; Saia et al., 2010).

Five of the studies give a description of the follow‐up period after the onset of BP or DS therapy. Saia et al. (2010) had a follow‐up period of 12 months, and after 3 months, four of five cases of MRONJ were reported, while the fifth case occurred after 6 months. In the study by Manfredi et al. (2017), the patients were followed for 60 months after the onset of medication and new cases of MRONJ were found up to 3 years after the onset of drug treatment. Yamazaki et al. (2012) had a follow‐up period of 42 months, while a follow‐up period of at least 12 weeks was applied by Bodem et al. (2015). Soutome et al. (2018) reported a follow‐up period of 8 years and MRONJ occurred up to 3 years after the start of BP therapy. The 1‐year cumulative occurrence rate for MRONJ was 8.6%, for 2 years 21.5% and for 3 years 29.2% calculated for all patients regardless of whether the teeth were extracted before or after medication start (Soutome et al., 2018).

### 3.4 Quality assessment

The summary assessment of the scientific quality (SBU, 2018) of the included studies showed that two studies had a low risk of bias (Hasegawa et al., 2019; Yamazaki et al., 2012) and one had a high risk (Walter et al., 2008). The other four studies had a moderate risk of bias. Quality ratings per study and outcome are shown in Figure 2. The quality evaluation using GRADE (Balshem et al., 2011) showed limited to moderately strong scientific evidence regarding the incidence of MRONJ and low evidence for all risk factors.

## 4 DISCUSSION

This systematic review investigated the incidence of bone necrosis (MRONJ) after tooth extractions in patients suffering from cancer and treated with high‐dose BP or DS. Tooth extraction is reported to be the most important risk factor for the emergence of MRONJ (McGowan et al., 2018). The incidence rates for MRONJ vary widely in this review from 11.4% to 50%. Two studies published after the search for this review was completed report a consistent incidence of MRONJ after tooth extractions of 28%–29% (Avishai et al., 2022; Hasegawa et al., 2021). The wide range in this review could partially be affected by the variation in follow‐up time. In addition to the tooth extraction itself, risk factors for developing MRONJ were studied. Teeth affected by inflammation before the extraction as well as surgical methods that meant that more jawbone was removed were identified as risk factors (Bodem et al., 2015; Hasegawa et al., 2019; Manfredi et al., 2017; Saia et al., 2010; Soutome et al., 2018; Yamazaki et al., 2012).

Since osteonecrosis caused by antiresorptive medication almost exclusively appears in the jaws, it has been discussed if the process is initiated by an odontogenic infection. Five of the included studies in this review reported that teeth with signs of inflammation, such as severe periodontal disease, osteomyelitis, or other symptoms of infection, before the treatment showed an increased risk of MRONJ after extraction (Hasegawa et al., 2019; Manfredi et al., 2017; Saia et al., 2010; Soutome et al., 2018; Yamazaki et al., 2012). The results therefore suggest that nonrestorable teeth and those with poor prognosis should be extracted before the development of inflammation and should not be postponed in cancer patients receiving high‐dose BP and DS. The observations differ from the results reported by Coello‐Suanzes et al. (2018), who could not describe connections between MRONJ and periodontal infection. However, in a Swedish prospective study with a 4‐year follow‐up, Hallmer et al. (2018) indicated that pre‐existing inflammatory dental disease such as periodontal disease or periapical pathology is a well‐recognized risk factor, assessed as the initiating factor for the development of MRONJ in 18% and 4% of the cases, respectively. Also, two recently published studies performed in Japan (Hasegawa et al., 2021) and Israel (Avishai et al., 2022) confirm that local inflammation processes present even before tooth extraction increase the risk of MRONJ. These findings can support the hypothesis that a periodontal infection in combination with antiresorptive treatment initiates osteonecrosis and not tooth extraction itself. However, it cannot be fully determined if the infection is in fact a primary or secondary event in the pathophysiology of MRONJ. Overall, the infection seems to be central to the process (Hallmer et al., 2018).

Nor did age or tobacco use show any relationship with the incidence of MRONJ, a result in line with the study by Hallmer et al. (2020). In a systematic review, the prevalence of medical and dental comorbidities reported from a total of 4106 cases of MRONJ was studied (McGowan et al., 2018). A total of 45% of the 4106 cases suffering from MRONJ registered tooth extraction as the most common risk factor, followed by periodontal disease (10%). Therefore, it is reasonable to suggest that optimizing the health of the oral cavity by improving oral hygiene, reducing inflammation, and treating infection to prevent the need for future invasive treatment should be an overarching aim of preventative dental care in risk patients.

Information on preventive measures is sparse in the included studies, which may be explained by the fact that most of the studies have a retrospective design. However, in the study by Manfredi et al. (2017), patients were offered oral hygiene recall and individual instructions for daily home oral hygiene. Those patients who started the dental program after the onset of BP therapy had a higher risk for MRONJ (Manfredi et al., 2017). Similar results have been reported by other studies (Coello‐Suanzes et al., 2018; Soutome et al., 2018). Beth‐Tasdogan et al. (2017) made the conclusion in a systematic review that individuals receiving high‐dose BP for advanced cancer and bone metastases should be placed on a regular recall schedule including a check of oral hygiene, periodontal diseases, cavities, and effective infection control.

In this review, relationships between medical conditions such as diabetes (Hasegawa et al., 2019; Soutome et al., 2018; Walter et al., 2008; Yamazaki et al., 2012) and the use of drugs such as cortico‐steroids and chemotherapy (Bodem et al., 2015; Hasegawa et al., 2019; Walter et al., 2008; Yamazaki et al., 2012) were analyzed in four studies without finding any significant relationships with the incidence of MRONJ. The results differ from the study by Hallmer et al. (2020), which reported a significantly decreased risk for MRONJ among patients who were medicated with corticosteroids. For those suffering from diabetes, a significantly increased risk for MRONJ was found, while no relationship between MRONJ and the use of chemotherapy could be registered (Hallmer et al., 2020). In a systematic review from 2018, the trends for reporting medical comorbidities related to MRONJ were shown. The risk of MRONJ associated with chemotherapy was described in scientific literature comprising more than 1400 patients but corticosteroids, cardiovascular diseases, and diabetes also occurred frequently (McGowan et al., 2018).

In this review, six studies reported the use of a combination of BP and DS, while in one study, only one type of BP was used (Manfredi et al., 2017). None of the studies reported significant differences in the incidence of MRONJ related to the specific drug. This differs from the study by Hallmer et al. (2018), who reported a prevalence of MRONJ of 1.03% when the patient used BP, compared to 3.64% for DS. In a recently published review, a meta‐analysis showed a prevalence of MRONJ between 4% and 19% depending on the type of used antiresorptive medication. An increased prevalence of MRONJ was noted in association with sequential therapy for pamidronate‐zoledronate and BP‐DS administration as compared to single therapy (Srivastava et al., 2021). Previous studies have shown that a longer duration appears to be associated with an increased risk (Avishai et al., 2022; Hasegawa et al., 2021). As reported by Ruggiero et al. (2014), regardless of indications for therapy, the duration of antiresorptive therapy continues to be considered a risk factor for developing MRONJ. One study in this review (Hasegawa et al., 2019) reported a significant increase in MRONJ when the duration of BP and DS medication exceeded 8 months. Intravenously administrated BP deposits effectively in bone tissues (Cremers et al., 2019). Long‐term use may therefore lead to high doses since BP deposits in the bone with a half‐life of 10–12 years, unlike DS which has a half‐life of 25 days (Damm & Jones, 2013). This disparity may make a difference in long‐term efficacy as well as adverse effects.

The undefined time period it takes to develop MRONJ leads to difficulties in deciding how long of a follow‐up period is required to find the real incidence of MRONJ. Hallmer et al. (2018) showed that patients on DS developed MRONJ after a mean duration of therapy of 16 months, while in patients on zoledronic acid, the mean duration time was 30 months. In a study by Henry et al. (2011), the incidence of developing MRONJ among cancer patients exposed to zoledronate or DS was 0.6% and 0.5%, respectively, after 1 year, 0.9% and 1.1%, respectively, after 2 years and 1.3% and 1.1%, respectively, after 3 years. Among DS‐exposed subjects, the risk for MRONJ achieved a plateau between Years 2 and 3 (Henry et al., 2011). Soutome et al. (2018) had a follow‐up period of 8 years with a cumulative occurrence rate of MRONJ of 8.6% after 1 year, 21.5% after 2 years, and 29.2% after 3 years. In the present review, Manfredi et al. (2017) had a follow‐up period of 60 months and new cases of MRONJ were detected 3 years after the onset of BP therapy. An MRONJ‐free survival at 3 years was reported to be 97% in a group having preventive dental treatments before the start of BP therapy. The corresponding proportion for those who received dental treatment after the onset of BP was 66% (Coello‐Suanzes et al., 2018). Theoretically, a low prevalence of MRONJ may be due to a short follow‐up period. It should therefore be preferable that studies of MRONJ incidence have a longer follow‐up period, such as 3–4 years. A large population (*N* = 3090) without antiresorptive therapy who had undergone extractions at a department of oral and maxillofacial surgery with a follow‐up period of 42 months showed an osteonecrosis incidence of 0.03% (Yamazaki et al., 2012).

Cancer patients receiving high‐dose antiresorptive medication such as BP or DS are often advised to avoid tooth extractions if possible or are recommended to temporarily discontinue the antiresorptive agents (drug holidays), which has been proposed to reduce the risk of MRONJ (Damm & Jones, 2013; Ruggiero et al., 2014). In the review by Ottesen et al. (2020), no evidence for using such a drug holiday with the purpose of decreasing the incidence of MRONJ could be found. A similar result was recently reported by Hasegawa et al. 2021). However, it should also be noted that due to a limited number of eligible patients, and a great variation between these patients, strong evidence for using drug holidays for high‐dose antiresorptive agents such as BP and DS is almost impossible to obtain. Therefore, when tooth extraction is necessary, in some countries, temporary discontinuation in treatment with antiresorptive drugs is still being recommended by national guidelines or position papers based on expert opinions. Due to the short half‐life, a temporary stop may have some benefits for DS, while a drug holiday when using BP may be ineffective owing to the long half‐life.

To prevent osteonecrosis after tooth extractions in patients treated with antiresorptive medications, an atraumatic surgical technique is recommended (Heufelder et al., 2014): use of antibiotics, mucoperiosteal flaps, bone edges smoothed, flaps prepared for tension‐free wound closure and sutures removed after 2 weeks. In this review, two studies reported that surgical processes could affect the outcome of MRONJ. Bodem et al. (2015) found that if osteotomy had to be performed during the surgical procedure, the prevalence of MRONJ significantly increased in comparison with extractions without osteotomy. Furthermore, Hasegawa et al. (2019) evaluated the surgical process and reported that root amputation increased the risk of MRONJ. As described in several other studies (Hallmer et al., 2018; Ruggiero et al., 2014), most cases of MRONJ were developed in the mandible, which is also the case in the studies included in this review. The surgical protocol was not a primary outcome in the present review and, in combination with the dominating retrospective study design, this may explain why the included studies usually lacked detailed descriptions of the surgical procedure.

In this systematic review, only studies that allowed a calculation of the incidence of MRONJ after tooth extractions in patients who were medicated with BP or DS were selected. All were observational studies and four had a control group. All included subjects had a malignant disease and received high‐dose intravenous medications. All studies reported a high and consistent incidence of MRONJ after tooth extractions. Most of the studies had a moderate risk of bias. At the same time, the follow‐up period varied widely, and long follow‐up periods showed that MRONJ may occur several years after the extraction was performed. Thus, regarding the incidence of MRONJ, the review shows limited to moderately strong scientific evidence. However, since only observational studies, combined with other study limitations, were available, the evidence was low for all risk factors. The low incidence of MRONJ and high variation among patients and antiresorptive therapies make it difficult to complete RCTs or controlled prospective studies with enough patients to be able to reliably answer the research question (Terpos et al., 2009).

## AUTHOR CONTRIBUTIONS

All authors contributed to the design of the study, analysis, and interpretation of the results and conclusions. All authors critically revised the manuscript, gave final approval, and agreed to be accountable for all aspects of the work.

## CONFLICT OF INTEREST

The authors declare no conflict of interest.

## Supporting information

Supporting information.

Supporting information.

## Data Availability

Data available in the article's Supporting Information.
