# Supplementary material for: Incidence and risk factors for medication‐related osteonecrosis after tooth extraction in cancer patients—A systematic review
Source: Clin Exp Dent Res. 2022 Dec 4;9(1):55–65. doi: 10.1002/cre2.698 (PMC9932256; doi:10.1002/cre2.698)
Supplement: Supplementary file 1 — Supporting information. [file CRE2-9-55-s001.docx]

**Incidence and risk factors for medication-related osteonecrosis after tooth extraction in cancer patients – A systematic review**

**Authors: Nurda Schwech, Johanna Nilsson, Pia Gabre**

Appendix, Table 1. Articles read in full text and which did not meet the inclusion criteria (n=36).

**Author(s) Title Journal**

Yasui et al. 2021 Influence of prostate cancer status on the prevalence Oral Surg Oral Med Oral

of medication-related osteonecrosis of the jaw Pathol Oral Radiol

Ueda et al. 2020 Does inflammatory dental disease affect the development Clin Oral Investig. of medication-related osteonecrosis of the jaw in patients

using high-dose bone-modifying agents?

Petrovic et al. 2019 Medication-Related Osteonecrosis of the Jaws: Two Biomed Res Int.

Center Retrospective Cohort Studies.

McGowan et al. Both non-surgical dental treatment and extractions increase Clin Oral Investig.

2019 the risk of medication-related osteonecrosis of the jaw:

case-control study.

Auzina et al. 2019 A population based study of multiple myeloma patients Stomatologija

with medication-related osteonecrosis of the jaw.

Egloff-Juras et al. Denosumab-related osteonecrosis of the jaw: A J Oral Pathol Med.

2018 retrospective study.

Coello-Suanzes et Preventive dental management of osteonecrosis of the Oral Dis.

al. 2018 jaws related to zoledronic acid treatment.

Ghidini et al. 2017 Medication-related osteonecrosis of the jaw: risk factors in Minerva Stomatol.

patients under biphosphonate versus patients under

antiresorptive-antiangiogenic drugs.

Matsumoto et al. Primary wound closure after tooth extraction for Clin Oral Investig.

2017 prevention of medication-related osteonecrosis of the

jaw in patients under denosumab.

Mücke et al. 2016 Prevention of bisphosphonate-related osteonecrosis of the J Craniomaxillofac Surg.

jaws in patients with prostate cancer treated with zoledronic

acid - A prospective study over 6 years.

Tardast et al. 2015 Bisphosphonate associated osteomyelitis of the jaw in J Appl Oral Sci.

patients with bony exposure: prevention, a new way of

thinking.

Rogers et al. 2015 United Kingdom nationwide study of avascular necrosis of Br J Oral Maxillofac Surg.

the jaws including bisphosphonate-related necrosis.

Otto et al. 2015 Tooth extraction in patients receiving oral or intravenous J Craniomaxillofac Surg.

bisphosphonate administration: A trigger for BRONJ

development?

Appendix, Table 1. Continue.

**Author(s) Title Journal**

Yuh et al. 2014 The national-scale cohort study on bisphosphonate-related J Dent.

osteonecrosis of the jaw in Taiwan.

Heufelder et al. Principles of oral surgery for prevention of Oral Surg Oral Med Oral

2014 bisphosphonate- related osteonecrosis of the jaw. Pathol Oral Radiol.

Verscovi et al. 2013 Case series of 589 tooth extractions in patients under Med Oral Patol Oral Cir Bucal.

bisphosphonates therapy. Proposal of a clinical protocol

supported by Nd:YAG low-level laser therapy.

Serrant and Clarke The characteristics of bisphosphonate patients developing Dent Update.

Et al. 2013 bisphosphonate-related osteonecrosis of the jaw attending

an OMFS department.

Kato et al. 2013 Evaluation of socket healing in patients undergoing Med Oral Patol Oral Cir Bucal.

bisphosphonate therapy: experience of a single Institution.

Thumbigere-Math A retrospective study evaluating frequency and risk factors Am J Clin Oncol.

et al. 2012 of osteonecrosis of the jaw in 576 cancer patients receiving

intravenous bisphosphonates.

Saad et al. 2012 Incidence, risk factors, and outcomes of osteonecrosis of Ann Oncol.

the jaw: integrated analysis from three blinded active-

controlled phase III trials in cancer patients with bone

metastases.

Mozatti et al. 2012 Platelet-rich therapies in the treatment of intravenous Oral Oncol.

bisphosphonate-related osteonecrosis of the jaw: a

report of 32 cases.

Miyazaki et al. 2012 Leukopenia as a risk factor for osteonecrosis of the jaw in BJU Int.

metastatic prostate cancer treated using zoledronic acid

and docetaxel.

Francini et al. 2011 Osteonecrosis of the jaw in patients with cancer who J Am Dent Assoc.

received zoledronic acid and bevacizumab.

Ferlito et al. 2011 Preventive protocol for tooth extractions in patients J Oral Maxillofac Surg.

treated with zoledronate: a case series.

Barash et al. 2011 Risk factors for osteonecrosis of the jaws: a case-control J Dent Res.

study from the CONDOR dental PBRN.

Walter et al. 2010 Prevalence of bisphosphonate associated osteonecrosis of Head Face Med.

the jaws in multiple myeloma patients.

Lodi et al. 2010 Tooth extraction in patients taking intravenous J Oral Maxillofac Surg.

bisphosphonates: a preventive protocol and case series.

Pavkovic et al. 2010 Osteonecrosis of the jaw in patients with multiple Prilozi.

myeloma treated with bisphosphanates.

Kyrgidis et al. 2008 Bisphosphonate-related osteonecrosis of the jaws: a case- J Clin Oncol.

control study of risk factors in breast cancer patients.

Appendix, Table 1. Continue.

**Author(s) Title Journal**

Hoff et al. 2008 Frequency and risk factors associated with osteonecrosis J Bone Miner Res.

of the jaw in cancer patients treated with intravenous

bisphosphonates.

Carmaguola et al. Dental and periodontal history of oncologic patients on Oral Surg Oral Med Oral

2008 parenteral bisphosphonates with or without osteonecrosis Pathol Oral Radiol Endod.

of the jaws: a pilot study.

Cafro et al. 2008 Osteonecrosis of the jaw in patients with multiple myeloma Clin Lymphoma Myeloma.

treated with bisphosphonates: definition and management

of the risk related to zoledronic acid.

Boonyapakorn et Bisphosphonate-induced osteonecrosis of the jaws: Oral Oncol.

al. 2008 prospective study of 80 patients with multiple myeloma

and other malignancies.

Walter et al. 2007 Prevalence of bisphosphonate associated osteonecrosis Support Care Cancer.

of the jaw within the field of osteonecrosis.

Mavrokokki et al. Nature and frequency of bisphosphonate-associated J Oral Maxillofac Surg.

2007 osteonecrosis of the jaws in Australia.

Capalbo et al. 2006 Jaw osteonecrosis associated with use of bisphosphonates Int J Hematol.

and chemotherapy: paradoxical complication of treatment

of bone lesions in multiple myeloma patients.
