# Supplementary material for: Incidence and risk factors for medication‐related osteonecrosis after tooth extraction in cancer patients—A systematic review
Source: Clin Exp Dent Res. 2022 Dec 4;9(1):55–65. doi: 10.1002/cre2.698 (PMC9932256; doi:10.1002/cre2.698)
Supplement: Supplementary file 2 — Supporting information. [file CRE2-9-55-s002.pdf]

Appendix Figure A

Figure 1. The search process leading to the search string for the data-base PubMed.

| Search Number | Search Term | Number of reference |
|---|---|---|
| 1 | Bisphosphonate-Associated Osteonecrosis of the Jaw[MeSH Terms] OR Alveolar Bone Loss[MeSH Terms] OR Bone Resorption[MeSH Terms] OR Osteonecrosis[MeSH Terms] | 55,235 |
| 2 | Bisphosphonate Associated Osteonecros*[Title/Abstract] OR Bisphosphonate-Associated Osteonecros*[Title /Abstract] OR Bisphosphonate-Induced Osteonecros*[Title/Abstract] OR Bisphosphonate Induced Osteonecros*[Title /Abstract] OR Bisphosphonate-Related Osteonecros*[Title/Abstract] OR Bisphosphonate Related Osteonecros*[Title /Abstract] OR Bisphosphonate Osteonecros*[Title/Abstract] OR "Medication-related osteonecrosis of the jaw"[Title /Abstract] OR MRONJ[Title/Abstract] OR BRONJ[Title/Abstract] OR "Alveolar Bone Loss*"[Title/Abstract] OR "Alveolar Resorption*"[Title/Abstract] OR "Periodontal Bone Loss*"[Title/Abstract] OR "Periodontal Resorption*"[Title /Abstract] OR "Alveolar Bone Atroph*"[Title/Abstract] OR "Bone Resorption*"[Title/Abstract] OR "Osteoclastic Bone Loss*"[Title/Abstract] OR Osteonecrosis[Title/Abstract] OR Osteonecroses[Title/Abstract] OR "Bone Necrosis"[Title /Abstract] OR "Bone Necroses"[Title/Abstract] OR "Avascular Necrosis of Bone"[Title/Abstract] OR "Bone Avascular Necrosis"[Title/Abstract] OR "Aseptic Necrosis of Bone"[Title/Abstract] OR "Bone Aseptic Necrosis"[Title/Abstract] OR Osteomyelitis[Title/Abstract] | 64,408 |
| 3 | 1 OR 2 | 98,946 |
| 4 | Tooth Extraction[MeSH Terms] | 20,478 |
| 5 | "Tooth Extraction*" [Title/Abstract] | 5,479 |
| 6 | 4 OR 5 | 22,760 |
| 7 | Bone Density Conservation Agents[MeSH Terms] OR Diphosphonates[MeSH Terms] OR Clodronic Acid[MeSH Terms] OR Zoledronic Acid[MeSH Terms] OR Ibandronic Acid[MeSH Terms] OR Anti-Bacterial Agents[MeSH Terms] OR Denosumab[MeSH Terms] OR Pamidronate[MeSH Terms] | 421,780 |
| 8 | "Bone Density Conservation Agent*"[Title/Abstract] OR "Antiresorptive Agent*"[Title/Abstract] OR "Antiresorptive Drug*"[Title/Abstract] OR "Antiresorptive treatment*"[Title/Abstract] OR "Bone Resorption Inhibitor*"[Title/Abstract] OR "Bone-modifying agent*"[Title/Abstract] OR "Bone Density Conservative Agent*"[Title/Abstract] OR Diphosphonate*[Title/Abstract] OR Bisphosphonate*[Title/Abstract] OR "Clodronic Acid"[Title/Abstract] OR "Dichloromethane Diphosphonate"[Title/Abstract] OR Dichloromethylenebisphosphonate[Title/Abstract] OR "Dichloromethanediphosphonic Acid"[Title/Abstract] OR Cl2MDP[Title/Abstract] OR Dichloromethanediphosphonate [Title/Abstract] OR Clodronate[Title/Abstract] OR Bonefos[Title/Abstract] OR "Zoledronic Acid"[Title/Abstract] OR "2-(Imidazol-1-yl)-1-hydroxyethylidene-1,1-bisphosphonic acid"[Title/Abstract] OR "CGP 42446A"[Title/Abstract] OR CGP-42446[Title/Abstract] OR "CGP 42446"[Title/Abstract] OR "CGP42446"[Title/Abstract] OR "CGP-42'446"[Title /Abstract] OR "CGP42'446"[Title/Abstract] OR "CGP 42'446"[Title/Abstract] OR Zometa[Title/Abstract] OR "Zoledronic Acid Anhydrous"[Title/Abstract] OR Zoledronate[Title/Abstract] OR "Ibandronic Acid"[Title/Abstract] OR Ibandronate[Title/Abstract] OR "1-Hydroxy-3-(methylpentylamino)propylidenebisphosphonate"[Title/Abstract] OR "(1-Hydroxy-3-(methylpentylamino)propylidene)bisphosphonate"[Title/Abstract] OR "Ibandronate Sodium"[Title/Abstract] OR Boniva[Title/Abstract] OR Bonviva[Title/Abstract] OR "RPR 102289A"[Title/Abstract] OR "RPR-102289A"[Title /Abstract] OR "RPR102289A"[Title/Abstract] OR Bondronat[Title/Abstract] OR "BM 21.0955"[Title/Abstract] OR "BM 210955"[Title/Abstract] OR "BM-21.0955"[Title/Abstract] OR "BM21.0955"[Title/Abstract] OR "BM-210955"[Title /Abstract] OR "BM210955"[Title/Abstract] OR "Anti Bacterial Agent*"[Title/Abstract] OR "Antibacterial Agent"[Title /Abstract] OR "Anti-Bacterial Agent*"[Title/Abstract] OR "Anti Bacterial Compound*"[Title/Abstract] OR "Antibacterial Compound*"[Title/Abstract] OR "Anti-Bacterial Compound*"[Title/Abstract] OR "Bacteriocidal Agent*"[Title/Abstract] OR Bacteriocide*[Title/Abstract] OR "Anti-Mycobacterial Agent*"[Title/Abstract] OR "Anti Mycobacterial Agent*"[Title /Abstract] OR "Antimycobacterial Agent*"[Title/Abstract] OR Antibiotic*[Title/Abstract] OR "Anticancer Agent*"[Title /Abstract] OR Antineoplastic*[Title/Abstract] OR "Antitumor Drug*"[Title/Abstract] OR "Antitumor Agent*"[Title /Abstract] OR "Cancer Chemotherapy Agent*"[Title/Abstract] OR "Cancer Chemotherapy Drug*"[Title/Abstract] OR "Chemotherapeutic Anticancer Agent*"[Title/Abstract] OR "Chemotherapeutic Anticancer Drug*"[Title/Abstract] OR Xgeva[Title/Abstract] OR "AMG 162"[Title/Abstract] OR Prolia[Title/Abstract] OR "Amino-1-hydroxypropane-1,1-diphosphonate"[Title/Abstract] OR "Amino 1 hydroxypropane 1,1 diphosphonate"[Title/Abstract] OR AHPrBP[Title /Abstract] OR Aminopropanehydroxydiphosphonate[Title/Abstract] OR Amidronate[Title/Abstract] OR "(3-Amino-1-hydroxypropylidene)-1,1-biphosphonate"[Title/Abstract] OR "Aminohydroxypropylidene Diphosphonate"[Title /Abstract] OR "1-Hydroxy-3-aminopropane-1,1-diphosphonic acid"[Title/Abstract] OR "1 Hydroxy 3 aminopropane 1,1 diphosphonic acid"[Title/Abstract] OR "Pamidronic Acid"[Title/Abstract] OR Pamidronate[Title/Abstract] OR Aredia [Title/Abstract] OR Steroids[Title/Abstract] OR Denosumab[Title/Abstract] | 544,166 |
| 9 | 7 OR 8 | 778,547 |
| 10 | Neoplasms[MeSH Terms] | 3,436,486 |
| 11 | Neoplasia*[Title/Abstract] OR Neoplasm*[Title/Abstract] OR Tumor*[Title/Abstract] OR Cancer*[Title/Abstract] OR Malignan*[Title/Abstract] | 3,171,484 |
| 12 | 10 OR 11 | 4,394,162 |

| | | |
|---|---|---|
| 13 | 3 AND 6 AND 9 AND 12 | 230 |
| | ((((Bisphosphonate-Associated Osteonecrosis of the Jaw[MeSH Terms] OR Alveolar Bone Loss[MeSH Terms] OR Bone Resorption[MeSH Terms] OR Osteonecrosis[MeSH Terms]) OR (Bisphosphonate Associated Osteonecros*[Title/Abstract] OR Bisphosphonate-Associated Osteonecros*[Title/Abstract] OR Bisphosphonate-Induced Osteonecros*[Title/Abstract] OR Bisphosphonate Induced Osteonecros*[Title/Abstract] OR Bisphosphonate-Related Osteonecros*[Title/Abstract] OR Bisphosphonate Related Osteonecros*[Title/Abstract] OR Bisphosphonate Osteonecros*[Title/Abstract] OR "Medication-related osteonecrosis of the jaw"[Title/Abstract] OR MRONJ[Title/Abstract] OR BRONJ[Title/Abstract] OR "Alveolar Bone Loss*"[Title/Abstract] OR "Alveolar Resorption*"[Title/Abstract] OR "Periodontal Bone Loss*"[Title/Abstract] OR "Periodontal Resorption*"[Title/Abstract] OR "Alveolar Bone Atroph*"[Title/Abstract] OR "Bone Resorption*"[Title/Abstract] OR "Osteoclastic Bone Loss*"[Title/Abstract] OR Osteonecrosis[Title/Abstract] OR Osteonecroses[Title/Abstract] OR "Bone Necrosis"[Title/Abstract] OR "Bone Necroses"[Title/Abstract] OR "Avascular Necrosis of Bone"[Title/Abstract] OR "Bone Avascular Necrosis"[Title/Abstract] OR "Aseptic Necrosis of Bone"[Title/Abstract] OR "Bone Aseptic Necrosis"[Title/Abstract] OR Osteomyelitis[Title/Abstract])) AND ((Tooth Extraction[MeSH Terms]) OR ("Tooth Extraction*"[Title/Abstract]))) AND ((Bone Density Conservation Agents[MeSH Terms] OR Diphosphonates[MeSH Terms] OR Clodronic Acid[MeSH Terms] OR Zoledronic Acid[MeSH Terms] OR Ibandronic Acid[MeSH Terms] OR Anti-Bacterial Agents[MeSH Terms] OR Denosumab[MeSH Terms] OR Pamidronate[MeSH Terms]) OR ("Bone Density Conservation Agent*"[Title/Abstract] OR "Antiresorptive Agent*"[Title/Abstract] OR "Antiresorptive Drug*"[Title/Abstract] OR "Antiresorptive treatment*"[Title/Abstract] OR "Bone Resorption Inhibitor*"[Title/Abstract] OR "Bone-modifying agent*"[Title/Abstract] OR "Bone Density Conservative Agent*"[Title/Abstract] OR Diphosphonate*[Title/Abstract] OR Bisphosphonate*[Title/Abstract] OR "Clodronic Acid"[Title/Abstract] OR "Dichloromethane Diphosphonate"[Title/Abstract] OR Dichloromethylenebisphosphonate[Title/Abstract] OR "Dichloromethanediphosphonic Acid"[Title/Abstract] OR Cl2MDP[Title/Abstract] OR Dichloromethanediphosphonate[Title/Abstract] OR Clodronate[Title/Abstract] OR Bonefos[Title/Abstract] OR "Zoledronic Acid"[Title/Abstract] OR "2-(Imidazol-1-yl)-1-hydroxyethylidene-1,1-bisphosphonic acid"[Title/Abstract] OR "CGP 42446A"[Title/Abstract] OR CGP-42446[Title/Abstract] OR "CGP 42446"[Title/Abstract] OR "CGP42446"[Title/Abstract] OR "CGP-42'446"[Title/Abstract] OR "CGP42'446"[Title/Abstract] OR "CGP 42'446"[Title/Abstract] OR Zometa[Title/Abstract] OR "Zoledronic Acid Anhydrous"[Title/Abstract] OR Zoledronate[Title/Abstract] OR "Ibandronic Acid"[Title/Abstract] OR Ibandronate[Title/Abstract] OR "1-Hydroxy-3-(methylpentylamino) propylidenebisphosphonate"[Title/Abstract] OR "(1-Hydroxy-3-(methylpentylamino)propylidene)bisphosphonate"[Title/Abstract] OR "Ibandronate Sodium"[Title/Abstract] OR Boniva[Title/Abstract] OR Bonviva[Title/Abstract] OR "RPR 102289A"[Title/Abstract] OR "RPR-102289A"[Title/Abstract] OR "RPR102289A"[Title/Abstract] OR Bondronat[Title/Abstract] OR "BM 21.0955"[Title/Abstract] OR "BM 210955"[Title/Abstract] OR "BM-21.0955"[Title/Abstract] OR "BM21.0955"[Title/Abstract] OR "BM-210955"[Title/Abstract] OR "BM210955"[Title/Abstract] OR "Anti Bacterial Agent*"[Title/Abstract] OR "Antibacterial Agent"[Title/Abstract] OR "Anti-Bacterial Agent*"[Title/Abstract] OR "Anti Bacterial Compound*"[Title/Abstract] OR "Antibacterial Compound*"[Title/Abstract] OR "Anti-Bacterial Compound*"[Title/Abstract] OR "Bacteriocidal Agent*"[Title/Abstract] OR Bacteriocide*[Title/Abstract] OR "Anti-Mycobacterial Agent*"[Title/Abstract] OR "Anti Mycobacterial Agent*"[Title/Abstract] OR "Antimycobacterial Agent*"[Title/Abstract] OR Antibiotic*[Title/Abstract] OR "Anticancer Agent*"[Title/Abstract] OR Antineoplastic*[Title/Abstract] OR "Antitumor Drug*"[Title/Abstract] OR "Antitumor Agent*"[Title/Abstract] OR "Cancer Chemotherapy Agent*"[Title/Abstract] OR "Cancer Chemotherapy Drug*"[Title/Abstract] OR "Chemotherapeutic Anticancer Agent*"[Title/Abstract] OR "Chemotherapeutic Anticancer Drug*"[Title/Abstract] OR Xgeva[Title/Abstract] OR "AMG 162"[Title/Abstract] OR Prolia[Title/Abstract] OR "Amino-1-hydroxypropane-1,1-diphosphonate"[Title/Abstract] OR "Amino 1 hydroxypropane 1,1 diphosphonate"[Title/Abstract] OR AHPrBP[Title/Abstract] OR Aminopropanehydroxydiphosphonate[Title/Abstract] OR Amidronate[Title/Abstract] OR "(3-Amino-1-hydroxypropylidene)-1,1-biphosphonate"[Title/Abstract] OR "Aminohydroxypropylidene Diphosphonate"[Title/Abstract] OR "1-Hydroxy-3-aminopropane-1,1-diphosphonic acid"[Title/Abstract] OR "1 Hydroxy 3 aminopropane 1,1 diphosphonic acid"[Title/Abstract] OR "Pamidronic Acid"[Title/Abstract] OR Pamidronate[Title/Abstract] OR Aredia[Title/Abstract] OR Steroids[Title/Abstract] OR Denosumab[Title/Abstract]))) AND ((Neoplasms[MeSH Terms]) OR (Neoplasia*[Title/Abstract] OR Neoplasm*[Title/Abstract] OR Tumor*[Title/Abstract] OR Cancer*[Title/Abstract] OR Malignan*[Title/Abstract])) | Search string |
